# Recent Advances in the Application Technologies of Surface Coatings for Fruits

**DOI:** 10.3390/foods14142471

**Published:** 2025-07-14

**Authors:** Limin Dai, Dong Luo, Changwei Li, Yuan Chen

**Affiliations:** School of Agricultural Engineering, Jiangsu University, Zhenjiang 212013, China; lmdai@ujs.edu.cn (L.D.); cwli@zju.edu.cn (C.L.)

**Keywords:** coating application technology, post-harvest preservation, microfluidic spinning, electrospinning

## Abstract

Globally, the proportion of the consumption of fruits in the human diet shows an increasing trend. However, fruits may incur significant losses during the post-harvest storage and transportation process due to metabolic activities and mechanical damage. Post-harvest coating technology has been proven to be an effective means of reducing quality loss, and it offers the advantages of being environmentally friendly, energy-efficient, and free of chemical residues. This article begins with an introduction to the three main mechanisms of coating preservation, including physical barrier effects, physiological metabolism regulation, and antibacterial and antioxidant effects. Secondly, this paper comprehensively reviews the latest progress of coating application technology in the field of fruit preservation, and summarizes the development of coating application technology in recent years, which is divided into two categories: traditional technology and fiber coating formation technology. Among these, the spraying method in traditional technology and microfluidic spinning technology in fiber coating formation technology are emphasized. This information will help to further develop coating application techniques to improve post-harvest fruit preservation.

## 1. Introduction

### 1.1. Background

As an important natural source of dietary fiber, vitamins, and minerals, fruits play an important role in maintaining human dietary balance, preventing micronutrient deficiencies, and reducing the risk of chronic non-communicable diseases [1]. According to the recommendations of the World Health Organization (WHO), an adult should consume about 400 g of fruits and vegetables per day [2]. According to the Food and Agriculture Organization of the United Nations (FAO), the world’s exports of major tropical fruits are expected to reach US$11 billion in 2024, a yearly increase of about 2%, accounting for an important position in global agricultural products [3]. Fresh fruits, on the other hand, usually have a high moisture content of 75–95% and maintain vigorous metabolic activity after harvest, which makes the fruit extremely perishable [4]. At the same time, in all agricultural supply chains around the world, post-harvest losses are more severe than natural spoilage due to mechanical damage, late harvesting, weather, and other factors during transportation, accounting for 28% to 55% of total production [5]. In South Asia, about 30% of fruits are lost each year due to inconvenient transportation and storage [6]. As a key link in the supply chain of agricultural products, post-harvest preservation technology has been continuously developed into various forms, such as refrigeration, radiation, modified atmosphere packaging, spraying antimicrobial agents and antioxidants, and adding microbial-derived preservatives [7,8,9,10,11,12]. However, each approach has limitations. For example, modified atmosphere packaging requires sophisticated equipment, refrigeration requires high operating and maintenance costs, and spraying antimicrobials and antioxidants produces toxic by-products or chemical residues [13,14].

Fruit coating technology effectively addresses these issues by applying one or several layers of edible material to the surface of the fruit, creating a physical barrier that hinders the movement of moisture, oxygen, and solutes, thereby slowing down oxidation and respiration, and extending the fruit’s shelf life [15]. This technology does not require complex equipment (refrigerated trucks or modified atmosphere storage) or high-energy consumption (refrigeration or a modified atmosphere), thus avoiding the potential residues of chemical preservatives and the controversies around consumer acceptance of radiation technology, while also being environmentally friendly and compatible [16,17]. Coating solutions are typically composed of various materials such as polysaccharides (chitosan, starch, cellulose), proteins (soy protein, casein), lipids (beeswax, paraffin), and composites [18,19,20,21,22]. In addition, the coating can also improve the appearance and luster of the fruit, which is more in line with consumers’ preference for natural, healthy preservation methods. Choosing the right coating method not only affects the coating effect formed on the surface of the fruit, but also affects the production cost and process efficiency. Therefore, this review discusses the latest progress of coating technology, clarifies its scope of application, advantages and disadvantages, and scalability, provides a reference for subsequent research and industrial application, and helps optimize the preservation process and reduce post-harvest loss.

### 1.2. The Mechanism of Action of Coating Preservation

**Figure 1:**
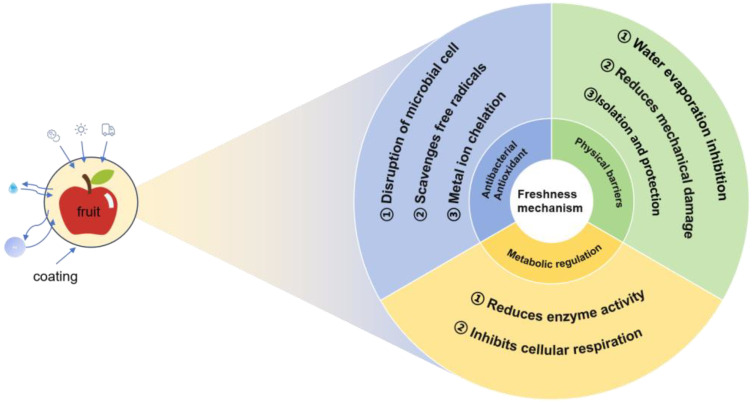
Preservation mechanism of fruit coating.

Fruits maintain an active physiological and metabolic state after harvest, and continuous respiration and transpiration can lead to quality deterioration such as water loss, tissue softening, and epidermal shrinkage. In addition, fruits are often subject to mechanical damage and bacterial infection during post-harvest storage and transportation, resulting in rapid fruit decay [23]. Therefore, as shown in Figure 1, the mechanism of fruit coating preservation was analyzed from three aspects: the physical barrier’s effect, the physiological metabolic regulation effect, and antibacterial and antioxidant effects. Understanding these basic preservation mechanisms is crucial for the rational selection and customization of coating application technologies to achieve the desired functional effects for specific fruits.

#### 1.2.1. Physical Barrier Effect

The physical barrier function of fruit coating for preservation is one of its core mechanisms, which restricts material exchange between the external environment and the fruit from multiple dimensions by forming a dense and continuous coating on the fruit [24]. From the perspective of moisture regulation, the sealing of stomata and cracks on the fruit’s surface reduces water vapor evaporation and maintains the internal moisture balance of the fruit [25]. From the perspective of mechanical protection, the coating fills the microcracks on the surface of the peel, enhances the resistance to mechanical damage, and minimizes the physical damage caused by collisions and compression during transportation or storage [26]. For example, Jiao et al. prepared a composite coating based on chitosan hydrochloride biguanide (CBg) and poly(N-vinylpyrrolidone) (PVP), which can be used as a fresh-keeping coating for strawberries. Due to the cross-linking between PVP and CBg, an interconnected network with low water vapor permeability is formed, significantly enhancing the coating’s flexibility and extensibility. When the CBg content is 5%, the water vapor transmission rate is 138.64 g/m^2^/day, and the Young’s modulus is 10.16 MPa [27].

#### 1.2.2. Physiological Metabolism Regulatory Functions

Physiological metabolism regulation delays fruit ripening and senescence while maintaining quality by intervening in key physiological processes such as post-harvest respiration, ethylene synthesis, enzyme activity, and secondary metabolites [28]. The coating inhibits gas exchange between the fruit and the external environment, reducing oxygen concentration and increasing carbon dioxide levels, thus forcing the fruit to transition from aerobic respiration to an inefficient anaerobic pathway, significantly decreasing respiratory entropy and energy metabolism rates, which in turn reduces the substrate supply for carbohydrate breakdown and ATP synthesis [29]. The active components loaded in the coating (such as o-phenylphenol) can specifically inhibit the activity of related enzymes in the respiratory chain, such as pyruvate decarboxylase and ethanol dehydrogenase, thereby reducing the accumulation of ethanol, a by-product of anaerobic respiration, and preventing cytotoxicity [30,31]. In addition, the reduction in respiration and enzymatic activity inhibits the expression of genes related to cell wall degradation, such as AcXETs, AcEXPs, and AcPE, decreases the activity of polygalacturonase, pectin methylesterase, and cellulase, inhibits cell wall degradation, and delays the post-harvest softening process. For instance, Liang et al. utilized chitosan and oxidized alginate as matrices to encapsulate cinnamaldehyde, preparing a composite coating with pH-responsive release properties. Under acidic conditions, cinnamaldehyde is selectively released, inhibiting the activity of key enzymes involved in ethylene biosynthesis. This mechanism can reduce ethylene production in lychees by over 50%, significantly delaying the decay process and maintaining fruit freshness and quality for over 8 days at room temperature [32].

#### 1.2.3. Antibacterial and Antioxidant Effect

The antibacterial and antioxidant efficacy of fruit coatings is achieved through multifaceted synergistic mechanisms. The antimicrobial action operates via three primary pathways: (1) the disruption of microbial cell membranes, (2) interference with nucleic acid metabolism, and (3) the chelation of essential metal ions [33]. For instance, chitosan and its derivatives, due to their positive charges, interact with the negatively charged components of bacterial cell film, thereby compromising membrane integrity and causing the leakage of intracellular substances. Additionally, low-molecular-weight chitosan can penetrate cells to interfere with DNA replication and transcription, inhibiting the metabolism of pathogens, while the chelation of metal ions (such as binding with Ca^2+^ and Mg^2+^) further obstructs the nutrients that are necessary for bacterial growth [34]. In terms of antioxidant action, it involves three approaches: scavenging free radicals, chelating metal ions, and activating enzyme activities [35]. For instance, the addition of natural ingredients (such as vitamin C and tea polyphenols) directly scavenges free radicals and chelates metal ions like Fe^2+^ and Cu^2+^ to inhibit the Fenton reaction, thereby reducing the accumulation of reactive oxygen species [36]. Simultaneously, the chitosan coating can also activate the endogenous antioxidant enzyme systems in fruits, such as superoxide dismutase and catalase, and decrease the generation of lipid peroxidation products like malondialdehyde [37]. Furthermore, the physical barrier function of the coating can reduce oxygen penetration, inhibit the oxidative metabolism caused by aerobic respiration, and block oxidative-inducing factors such as ultraviolet rays. Liu et al. prepared tannic acid/MXene assembly with chitosan (CS-TA/MXene) composite membranes. The physical slicing of MXene synergizes with the phenolic hydroxyl groups of TA to enhance antimicrobial efficacy, achieving a bactericidal rate of 80% within 6 h, whereas pure CS membranes only reach 20%. Additionally, the phenolic hydroxyl groups of TA serve as the primary contributors to antioxidant activity, enabling the CS-TA/MXene composite membrane to achieve a DPPH radical scavenging rate of 89.6%, demonstrating excellent antioxidant properties. In contrast, the pure CS membrane only achieved 30.2% [38].

Antioxidation is one of the core mechanisms for preserving freshness in coatings, and improving antioxidant performance can enhance preservation effects [39]. Controlled-release technology can extend the effective concentration time of antioxidants, delaying fruit spoilage, and is widely applied. However, research has shown that in lipid models, using high concentrations of antioxidants in the early stages of oxidation is more effective than using moderate to low concentrations in the later stages [40]. Lipid oxidation increases exponentially in the early stages, and if not inhibited early on, once it reaches the proliferation stage, this trend cannot be prevented. This suggests that in high-fat fruits such as avocados and coconuts, complete addition may be more effective than controlled release.

## 2. Application Techniques of Traditional Coatings

**Figure 2:**
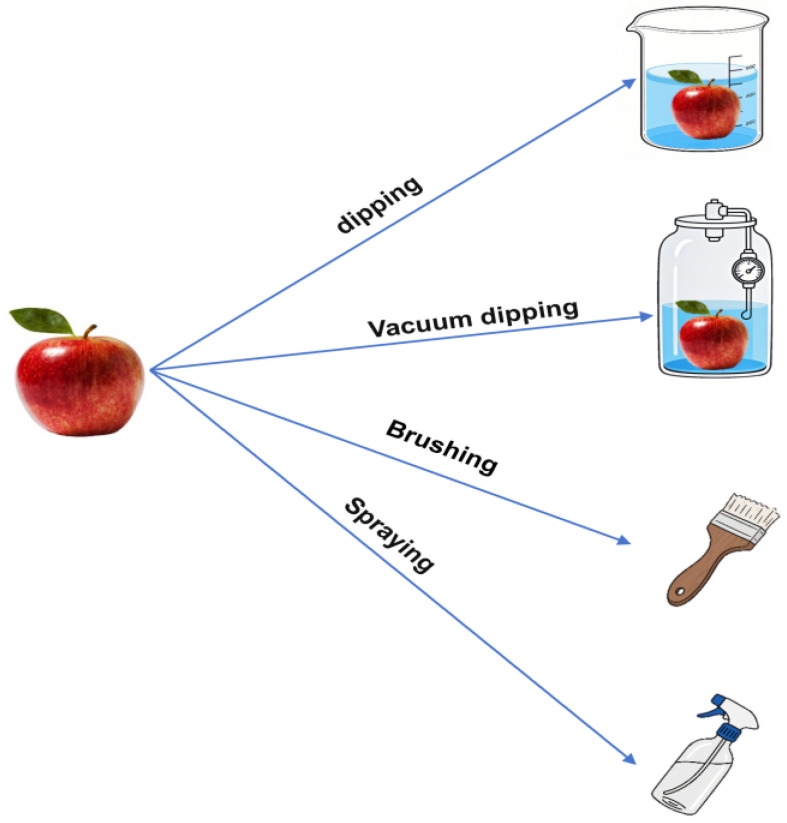
Traditional coating techniques.

As shown in Figure 2, traditional coating application techniques include dipping, spraying, and brushing. Vacuum dipping is developed based on dipping, and different coating techniques are selected according to the types of fruits, surface properties, and other factors. Table 1 demonstrates the coating techniques for different fruits [41].

Table 2 summarizes the advantages and disadvantages of traditional coating technologies from the perspectives of performance, economy, operability, and applicability. Currently, the most commonly used application methods for fruit coatings are immersion and spraying methods, while the brushing method is gradually being eliminated from the market due to its inferior effectiveness.

### 2.1. Dipping

The dipping method, recognized as one of the most ancient and widely utilized physical coating techniques, traces its origins to the 12th–13th century in China, where wax coatings were applied to fresh citrus fruits and lemons to prevent desiccation [55]. To date, this method remains extensively employed in fruit preservation, operating on the principle of immersing fruits into a pre-formulated coating solution. During immersion, solutes adsorb onto and deposit across the fruit surface, ultimately forming a continuous protective layer. The process involves three sequential steps: immersion, deposition, and drying [56]. In the first step, the fruit is evenly and slowly placed in a solution containing a coating-forming matrix and soaked for a certain amount of time (usually 30 s to several minutes) to ensure that the solute fully penetrates the surface of the peel. In the second step, the fruit is taken out and allowed to drain naturally, with excess solution removed by gravity, at which point the solute gradually accumulates in the micropores or epidermal structure of the peel. In the third step, it is dried under ambient or low-temperature ventilation to promote the volatilization of the solvent or the cross-linking and solidification of the solute, forming a continuous, dense, semi-permeable membrane [57]. Repeating the above steps can achieve multi-layer coatings, and if different coating solutions are alternately overcoated, coatings with different functions can be obtained [58]. The quality characteristics of the coating depend on several factors. From an operational perspective, this includes the number of overcoating cycles, the speed at which the fruit is removed from the solution, and the duration of the soaking. From the point of view of coating solution properties, this includes density, viscosity, and surface tension [55,59]. In addition, drying conditions and fruit surface properties also play an important role in coating density and morphology. It should be noted that the impregnation method has the following problems. Firstly, the coating will be diluted during operation, and at the same time, waste or dirt is easy to accumulate during the impregnation process, and microorganisms may grow in the impregnation tank. Secondly, from the point of view of the impregnation mechanism itself, it has certain defects, and after the impregnation process, the natural wax coating on the original surface of fruits and vegetables is likely to peel off due to the solvent properties of the impregnation process and the physical actions (dissolution, erosion, stress) during the operation [60].

### 2.2. Vacuum Dipping

The vacuum dipping method is an advanced technology of the dipping method, which has something in common with the ordinary dipping method in operation. The core difference is that, in the fruit maceration process, the vacuum impregnation technology is carried out in a vacuum environment. In the maceration process, the fruit is immersed in an airtight vacuum chamber connected to a vacuum pump, through which the air in the impregnation tank is evacuated, and the impregnation solution is driven by a negative pressure difference to penetrate the pores or micropores on the surface of the fruit, usually for 10–30 min, and the pressure depends on the type of fruit [61]. After vacuum dipping, we slowly open the inlet valve of the vacuum tank, so that the outside air gradually enters the vacuum tank, it slowly returns to the normal pressure state, and the fruit remains immersed in the coating solution at atmospheric pressure. In this process, it is necessary to control the air intake speed to avoid rapid pressure changes that may cause damage to the fruit tissue or uneven coating [62]. Compared with dipping, vacuum dipping inhibits microorganisms and oxidative enzymes better under vacuum conditions, preserves the natural flavor and nutritional value of fruits, and further extends the shelf life of fruits. It has been shown that it can be used in some fruits, such as mango, strawberry, apricot, and papaya [63,64]. However, the application of this technique on fruits is mostly used for penetrating dehydration rather than coating fresh fruits.

### 2.3. Brushing

The brushing method is a technique that involves applying a protective coating to the surface of a fruit by using a roller, a cloth cover, and a soft brush dipped in the coating solution [41]. The bristles are adaptable, especially for fruits with irregular or sunken skins (e.g., strawberries, lychees), and can be manually adjusted to reduce the risk of missed application [65]. In addition, the coating quality can be enhanced by targeting localized damage areas (e.g., at the apple stalk). The coating quality of brush application usually depends on factors such as the type of brushes, brushing strength, and brushing time. It was found that horsehair brushes gave better results after coating compared to nylon brushes [66]. The percentage of different materials in the same type of brush also affects the brushing and coating effect, for example, changes in the ratio of natural to synthetic bristles will change the amount of adsorption, elasticity, or durability of the bristles, which affects the uniformity, thickness, or smoothness of the coating, and the higher the percentage of the material, the more significant its dominant characteristics will be during the brushing and coating process [67].

### 2.4. Spraying

Spraying is the most widely used method for fruit coatings, dispersing a fine layer of liquid coating through a nozzle onto the surface of the fruit. At present, three different spraying technologies, namely pressure atomization, air spray atomization and air-assisted airless atomization, are applied to fruit coatings [68].

Pressure atomization involves pressurizing the coating liquid through a high-pressure pump, forcing the liquid to be sprayed out of the nozzle at a high speed under high pressure, and the pressure energy is converted into kinetic energy, which is directly atomized into fine particles after overcoming the surface tension. Since there is no air involved in this process, pressure atomization is also called airless atomization. Pressure atomization typically uses small nozzles and is suitable for high-viscosity coatings and can form thicker coatings [69].

Air atomization involves using high-speed compressed air to impact a liquid, breaking down its surface tension and viscosity. Through shear action, the liquid is broken into mist-like particles, and the mist droplets flow through the nozzle outlet with the airflow to form a spray. The spray width and mist droplet distribution can be precisely controlled by adjusting the nozzle diameter, airflow angle, and spray distance. This spraying method is uniform and suitable for low-viscosity coatings, but the coating utilization rate is relatively low [70]. Wladimir Silva-Vera et al. formulated coatings based on hydroxypropyl methylcellulose, k-carrageenan, glycerol, and cellulose nanofibers, which were sprayed onto grape surfaces under conditions of flow rates of 1 or 5 L·h^−1^, pressures of 50 or 200 kPa, and heights of 0.3 or 0.5 m. The optimal conditions were a suspension flow rate of 1 L·h^−1^, an air pressure of 200 kPa, and a nozzle height of 0.5 m [71].

Air-assisted airless atomization is a technology that integrates the advantages of airless atomization and air atomization. Its core principle is to achieve precise atomization through the synergistic effect of high-pressure liquid kinetic energy and low-pressure gas assistance [72]. Firstly, a high-pressure pump pressurizes the coating liquid to a supercritical state, creating a high-speed jet through a special nozzle to complete the initial atomization. Subsequently, low-pressure air is introduced around the nozzle, which breaks down the coarse droplets generated by the initial atomization through the shear force at the gas–liquid interface, ultimately forming a mist cloud with a uniform particle size and a dense distribution [73]. This dual energy release mechanism retains the adaptability of airless atomization for high-viscosity liquids while optimizing atomization efficiency through air assistance, significantly reducing droplet drift loss, and is especially suitable for the uniform coating needs of fruits and vegetables with irregular surfaces [74]. Air-assisted airless atomization solves many of the challenges faced in the use of high-viscosity, high-solid coatings, and also overcomes a series of problems caused by heating and the use of excessive fluid pressure to atomize viscous materials. This method not only achieves a high level of production, but also ensures a high-quality surface treatment.

When spraying, the surface of the liquid oscillates and is disturbed. This is because the cohesive force tries to maintain the liquid’s original aggregation state, while the destructive force competes with it to break up the liquid into small droplets and push it to attach to the surface of the fruit [75]. Only when these two forces are properly balanced can the coating material form a uniform, stable, and good adhesive coating on the fruit surface [76]. During the spraying process, the parameters that affect the spray effect include the atomization pressure of the coating solution, viscosity, spray thickness, surface temperature and tension, as well as the shape and design of the nozzle [77,78,79]. Atomization pressure is a critical parameter in spraying technology, and research has found that maintaining the surface pressure of starch-methylcellulose membranes below 3.5 bar can protect the coating-forming process from being disrupted [80]. Additionally, the water vapor and mechanical properties are at their best with a coating thickness of 30 μm, making it crucial to control this parameter [81]. Sana Yakoubi et al. utilized machine learning techniques to predict and optimize spraying conditions to enhance the performance of edible coatings on plantain peel, and identified the optimal coating conditions, an air pressure of 0.6 MPa, a height of 0.15 m, and a time of 5 s, achieving a coating thickness of 38.5 μm [82].

Compared with the above three traditional technologies that rely on atomized liquid to achieve coating, electrostatic spraying, as an emerging spraying technology, can make the paint droplets charge and directionally adsorb onto the target surface through a high-voltage electrostatic field, which can flexibly adjust the flow rate and solution viscosity according to actual needs, customize the droplet specifications, and achieve accurate control of the thickness and uniformity of the coating [83,84]. The effectiveness of electrostatic spraying is affected by various factors, including the percentage of solvent in the solution, and the solution’s viscosity, conductivity, voltage, receiving distance and flow rate [85]. While conventional spraying solutions can form droplet size distributions of up to 20 μm, electrostatic spraying technology can generate uniform particles with a size of less than 100 nm from polymer and biopolymer solutions. Currently, electrostatic spraying has been implemented on some fruit coatings. Greta Peretto et al. employed electrostatic spraying technology as an innovative and efficient method to apply an edible alginate coating that is rich in carvacrol and methyl cinnamate to fresh strawberries. A comparison was made between the efficiencies of electrostatic spray coating technology and traditional spray coating technology in terms of transfer efficiency and coating uniformity. The electrostatic spray coating technology demonstrated higher transfer efficiency and uniformity than the traditional spray coating technology, with the delay in microbial spoilage being greater (11 days) compared to the traditional spray coating technology (10 days) [68]. In addition, when constructing multi-layer coatings, the spray method is similar to the dip coating method in terms of the operating process, and the multi-layer coating can be built layer by layer by spraying and drying multiple times.

It is worth noting that some researchers have applied dip coating, brush coating, spray coating, and electrostatic spray coating to mangoes. The study found that spray coating and electrostatic spray coating yielded superior combined results, with nearly identical preservation capabilities. Mangoes treated with dip coating maintained the highest hardness and lowest weight loss rate. Electrostatic spraying produces thinner coatings, shorter drying times, and higher transfer efficiency. However, electrostatic spraying can reduce paint consumption, reduce reliance on manual labor, and achieve automation in the coating process, making it more promising for future development [86]. Extending this research to different fruits to further validate the advantages of electrostatic spraying is a good approach.

## 3. Fiber Coating-Forming Technology

**Figure 3:**
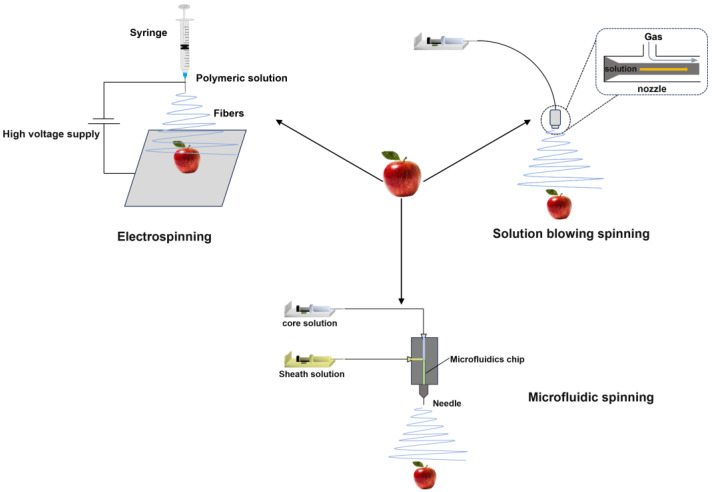
Fiber coating forming technology.

Although traditional coating methods are widely used, traditional coating technologies face challenges such as coating uniformity, adhesion strength, and limited control over film microstructure. Their preservation effects cannot fully meet requirements, so it is crucial to explore effective preservation strategies to maintain fruit quality during storage [87]. In recent years, the preparation of functional nanofiber membranes has attracted much attention in the field of fruit preservation, and nanofiber membranes have the characteristics of a large specific surface area, high porosity, good biocompatibility and biodegradability, and excellent mechanical properties, and can flexibly adjust the shape, size, and performance of nanofiber membranes according to different application needs [88,89,90]. This type of coating can form a barrier on the surface of the fruit, effectively preventing the invasion of harmful substances and microorganisms, and the nanofiber coating can be further functionalized with antibacterial, antioxidant, conductive, and photocatalytic and other functions [91,92]. As shown in Figure 3, after years of research, three types of fiber coating-forming technologies have been developed: electrospinning, solution-blowing spinning, and microfluidic spinning. Additionally, Table 3 illustrates the selection of fiber coating-forming techniques for different fruits.

### 3.1. Electrospinning Technology

#### 3.1.1. Principle of Electrospinning

The electrospinning process, which typically consists of a high-voltage power supply, a spinneret, a constant-current syringe pump, and a receiving device (current collector), is a technology that uses a high-voltage electrostatic field to process a polymer solution into nano- to microfibers. The basic principle of electrospinning is based on the synergistic effect of the electric field force and surface tension. Under the influence of a high-voltage electrostatic field, the polymer solution gradually forms a “Taylor cone” structure on the needle [99,100].

As the electric field force continues to increase, the original droplet-shaped solution is stretched and transformed into a charged jet, which is sprayed in the direction of the receiving plate [101]. During this process, the jet is influenced by various interaction forces, with electrostatic repulsion being particularly crucial, along with other interactions. These forces work together to cause the jet to break up and refine as the solvent continuously evaporates, ultimately forming a nanofiber membrane on the receiving plate [102]. Electrospinning technology is applicable to various polymer materials, such as natural polymers (like collagen and chitosan) and synthetic polymers (such as polylactic acid and polystyrene), and even inorganic nanoparticles (like titanium dioxide and zinc oxide) can be uniformly dispersed in polymer fibers to produce composite nanofibers with special functions [103,104,105,106]. Compared to traditional techniques, electrostatic spinning can produce nanofibers with diameters of less than 1 mm, while enabling them to obtain nanoscale structures [107,108].

#### 3.1.2. Classification and Application of Electrospinning

Based on the differences in spinning solutions and needles, electrospinning can be further classified into three distinct categories: uniaxial electrospinning, coaxial electrospinning, and emulsion electrospinning.

Uniaxial electrospinning is the most widely used technology, which uses a single nozzle, and the spinning solution is usually a single polymer solution or a blending solution formed by uniformly mixing multiple polymers and additives [109]. Because its solution is often a mixture, it is also referred to as uniaxial (mixed) electrospinning [110]. Uniaxial nanofibers can encapsulate active molecules within the fibers, thereby achieving the sustainable release of these active molecules. Furthermore, by adding functional nanoparticles, the toxic effects on cells can be effectively reduced, minimizing cytotoxicity. Beyza Sukran Isik et al. encapsulated the polyphenols from tart cherry concentrate with gelatin or gelatin-whey protein through uniaxial electrospinning, resulting in an 8-fold increase in the protective effect on cyanidin-3-glucoside by electrospinning compared to the unencapsulated tart cherry concentrate [111]. Nevertheless, uniaxial nanofibers have limitations in preservation coatings, as the bioactive compounds encapsulated within them are easily disrupted by environmental factors, leading to the potential sudden release of active molecules, which makes it challenging to meet the requirements for the stability and sustained action of active molecules in fruit preservation [99].

Compared to uniaxial electrospinning, coaxial electrospinning synchronously extrudes two or more immiscible solutions through a specially designed coaxial nozzle, creating nanofibers with complex structures such as ‘core–shell’, hollow, and multi-channel structures [112]. The release of bioactive compounds is one of the main actions hindering the widespread application of electrospinning nanofiber materials, and coaxial electrospinning effectively provides a viable solution by forming a special ‘core–shell’ structure [113]. The shell layer of the core–shell structure (such as polymers or hydrophobic materials) can isolate the external environment (humidity, oxygen, ultraviolet light, etc.) and protect the sensitive active ingredients (such as antimicrobial agents, antioxidants, enzymes, etc.) within the core layer. The core layer of the core–shell structure regulates the release of active ingredients in the core through the degradation rate or porosity of the shell material [114]. The two complement each other to ensure the efficient utilization of active ingredients and adaptation to complex environments. Zhang et al. successfully prepared a biodegradable core–shell nanofiber membrane loaded with thymol for strawberry preservation through a coaxial electrospinning process. Their research found that it effectively inhibited the growth of bacteria, fungi, and yeast, extending the shelf life of the fruits [115].

Emulsion electrospinning is a method for preparing nanofibers with core–shell or porous structures by forming an emulsion from two immiscible liquids, combined with electrospinning technology [116]. Its key feature is that it does not require a complex nozzle apparatus and functional components can be encapsulated using a single nozzle. Compared to traditional coaxial electrospinning, emulsion electrospinning offers significant advantages, such as the encapsulation of hydrophobic bioactive molecules within the core structure of nanofibers, but selecting the appropriate polymer and emulsion parameters remains a major challenge in this field [117]. Cui et al. prepared a PVA/PCL-citral nanofiber membrane for apricot preservation using emulsion electrostatic spinning technology, and the experimental results showed that the free radical scavenging rate was 88%, the antimicrobial rates of *Escherichia* coli and *Staphylococcus* aureus were 99.99% and 99.98%, respectively, and the shelf-life was prolonged up to 9 days, which showed good antimicrobial and antioxidant properties [118].

#### 3.1.3. Factors Influencing Electrospinning

The effect of electrospinning coatings is closely related to the diameter and morphology of the nanofibers and is largely constrained by the characteristics of the electrospinning polymer solution, the spinning process parameters, and the surrounding environmental conditions [119]. To achieve the best application performance of nanofibers, it is essential to identify the most appropriate electrospinning parameters and produce fibers with uniform diameters and an intact morphology. In fact, electrospinning, as a technique for encapsulating active compounds, can achieve greater encapsulation efficiency for those compounds by optimizing the spinning process parameters compared to merely improving the properties of the polymer solution and the surrounding environmental conditions [120]. Therefore, optimizing the conditions for electrospinning is a more efficient and economical strategy.

### 3.2. Solution-Blowing Spinning Technology

Solution-blowing spinning has been developed based on traditional electrospinning technology, and common equipment includes syringe pumps, custom nozzles, high-speed gas sources (usually air), and collectors [121]. It ejects the polymer solution from the nozzle using compressed air or other gases. Under the influence of high-velocity airflow, the solution is stretched into filaments while the solvent evaporates during flight, ultimately forming a nanofiber felt or other specific structures of nanofiber materials on the collection device [122]. Compared to electrospinning, solution-blowing spinning technology has the advantages of a higher production efficiency, lower costs, and no need for high-voltage power supplies, allowing for the preparation of a large quantity of nanofibers in a short time [123]. At the same time, it has relatively low requirements for the properties of polymer solutions, and can be applied to a wider variety of polymers, including some nanofibers that are difficult to produce using electrospinning [124]. In addition, solution-blowing technology can also precisely control the diameter, shape, and orientation of nanofibers by adjusting process parameters to meet the needs of different application areas [125].

In solution-blowing spinning technology, fiber morphology and properties are influenced by the following factors: solution variables (polymer concentration and molar mass, viscosity, surface tension, solvent type, particles or additives), processing parameters (solution feed rate, gas pressure, nozzle-collector distance), the equipment configuration (nozzle diameter, nozzle geometry), and environmental parameters (temperature, humidity, atmospheric pressure) [126,127,128,129]. As an emerging technology, there is a scarcity of relevant data regarding the prediction and control of fiber diameter during the blow spinning process. Although some studies have outlined the general mechanisms of fiber formation and discussed the influence of certain processing parameters on fiber morphology, they remain insufficient to support precise control of this technology in critical aspects such as fiber diameter regulation [130]. Currently, research has demonstrated that solution-blowing technology can be used for fruit preservation coatings. Shen et al. quickly prepared nanofiber films composed of chitosan/polycaprolactone loaded with thymol/2-hydroxypropyl-β-cyclodextrin inclusion complexes using solution-blowing technology. The developed films achieved a sustained release of thymol over 240 h and exhibited significant antifungal activity [124]. Hann et al. prepared polyvinyl alcohol nanofiber membranes containing aqueous extracts of acai berry pulp, cocoa shell, jabuticaba peel, and carrot residue via solution-blowing spinning. The results showed that the nanofibers exhibited a uniform morphology with diameters ranging from 352 to 504 nm, induced minimal fruit color change, reduced antioxidant activity and TPC degradation by over 30%, and decreased fruit deterioration incidence by 50% [96].

### 3.3. Microfluidic Spinning Technology

Microfluidic spinning technology is a cutting-edge approach that integrates microfluidic technology with traditional spinning processes, allowing for the precise control of fluids through micron-scale channels to produce nanofibers with complex structural and functional characteristics [131]. In this technology, fibrillating polymers are the core raw materials, usually injected as core flows into microchannels, which can solidify or phase-separate under specific conditions to form fibers. The sheath flow is generally an immiscible fluid that flows around the core flow, controlling the size, morphology, and structure of the fibers through the constraints, stretching, and curing of the core flow. Traditional spinning technologies (electrospinning, dry spinning, wet spinning) require high temperatures, high pressures, or toxic reagents to operate. This not only increases costs and risks, but also makes it difficult to meet the safety and environmental requirements for fruit preservation, greatly limiting its applications in this field [132]. In microfluidic spinning technology, factors such as flow rate, channel size, and type are easily controlled, allowing for the easy production of fibers with varying microstructures and uniform sizes [133]. This technology has garnered significant attention in the field of fruit preservation coatings due to its mild reaction conditions, excellent mechanical properties, and ability to control fiber configuration.

The microfluidic spinning device mainly consists of three parts: the spinning liquid injection device, the microfluidic chip, and the fiber collection device. As the core component, the microfluidic chip controls the fluid behavior through microchannel designs (such as Y-shaped, T-shaped, and coaxial designs) [134]. Currently, the materials used to fabricate microfluidic chips are mainly categorized into the following types: inorganic materials, organic materials, and composite materials. Silicon and glass are the two most commonly used inorganic materials for chips, which possess a high surface stability, adjustable thermal conductivity, and solvent compatibility, but it is challenging to produce high specific surface areas and anisotropic structures [135]. Compared to inorganic materials, the selection of organic materials is more diverse, including polystyrene (PS), polymethyl methacrylate (PMMA), and polycarbonate (PC). They have advantages such as lower costs, and a faster and simpler fluid rotation process, but they perform poorly in terms of aging resistance, chemical resistance, and mechanical properties [136]. Composites achieve high mechanical strength, biocompatibility, and functional integration through the combination of multiple materials. However, the processing techniques are complex, and differences in thermal expansion coefficients among the various materials may lead to structural stability issues [137,138].

#### 3.3.1. Solidification Methods for Microfluidic Spinning Fibers

The current methods for fiber curing mainly include four types: solvent evaporation, solvent exchange, ionic cross-linking reactions, and chemical cross-linking reactions [132].

The chemical cross-linking reaction is a widely used method for curing microfluidic spinning fibers, utilizing microfluidic chips as microreactors to mix reactants during the spinning process and undergo chemical reactions, forming a three-dimensional network structure through covalent bonds, which significantly enhances the mechanical properties of the fibers and prevents them from dissolving or deforming during subsequent processing [139]. CA, GA, and TA are frequently used as chemical cross-linking agents [140,141,142]. He et al. utilized a chitosan aqueous solution as the core flow and GA as the sheath flow to prepare chitosan tubular fibers with excellent mechanical properties [143].

An ionic cross-linking reaction is also commonly used for curing microfluidic spinning fibers, which is roughly similar to the chemical cross-linking reaction mechanism [99]. Ion cross-linking is often used in microfluidic spinning for natural polymers such as sodium alginate and chitosan, because these materials have good biocompatibility and fast reaction properties [144,145]. Hu et al. used microfluidic spinning technology to prepare alginate fibers with enhanced mechanical properties by employing an alginate solution as the core flow and a CaCl_2_ solution as the crosslinker [146].

Solvent exchange is a method used to solidify microfluidic spinning, usually with the fiber-forming polymer solution as the core stream and the coagulant reagent as the sheath stream. When the two solutions are in contact within the microchannel, the fiber-forming polymer solution rapidly solidifies into fibers. Therefore, the choice of fiber-forming polymer solution and coagulant reagent directly affects the quality of fibers. Ethanol, methanol, and water are usually used as coagulant reagents, while chitosan, PCL, and PVA are usually used as fiber-forming polymer solutions [147,148,149]. Traditional microfluidic spinning to construct fibers is usually based on the principle of ionic cross-linking or chemical cross-linking, which is subject to the limitations of raw materials (sodium alginate, PEGDA, dextran, etc.), resulting in low product strength and limiting development and application. Liu et al. developed a novel microfluidic spinning method based on dual-solvent phase transfer principles for constructing high-strength helical fibers. Their approach demonstrated broad applicability across multiple polymers, including polycaprolactone (PCL), polyvinyl butyral (PVB), polysulfone (PSF), and polyethersulfone (PES) [150].

Solvent evaporation is also a common method used to solidify microfluidic spinning, where the solvent volatilizes in air or a heated environment, increasing the concentration of the polymer and solidifying into fibers. The microfluidic spinning device usually has only one microchannel, and the direct use of a polymer solution to prepare fibers will lead to poor mechanical quality, so it is necessary to mix crosslinkers with polymer solutions or blend multiple solutions to improve the performance of microfluidly spun fibers [151]. It is found that sodium polyacrylate (PAAS), polymethyl methacrylate (PMMA), and polyvinylpyrrolidone (PVP) can significantly improve the mechanical properties of fibers when used as crosslinking agents [152,153]. The effect of solvent evaporation curing fibers is mainly affected by the evaporation rate, which is affected by the nature of the solvent, ambient temperature and humidity, and airflow control and other factors [154,155].

#### 3.3.2. Application of Microfluidic Spinning in Fruit Coatings

Microfluidic spinning technology has attracted much attention in the field of fruit preservation coating because of its advantages in its rapid preparation of nanofiber membranes, precise control of the spinning process, and direct in situ formation of nanofiber membranes on different fruit surfaces [156,157]. At present, microfluidic spinning technology is mainly studied on fruit coatings, and microfluidic blow spinning is developed on the basis of blow molding spinning, which is an innovative method combining traditional blow molding spinning and microfluidic technology, and has significantly improved in terms of structural diversification, preparation efficiency, and mechanical properties [158]. In recent years, the research on microfluidic blow spinning in fruit coating has mostly improved the preservation effect of the coating by loading antibacterial molecules and blending solutions, and the main materials of the coating solution include polycaprolactone (PCL), konjac glucomannan (KGM), alginate, and chitosan [132]. Lin et al. combined konjac glucomannan (KGM) with elderberry anthocyanin (EA) to form a coating solution (KEA), blended it with a polyvinylpyrrolidone (PVP) solution, and prepared KEA/PVP fiber coatings via microfluidic blown film technology. Experimental results demonstrated excellent thermal stability, water vapor barrier properties, and mechanical strength, with DPPH and ABTS free radical scavenging rates reaching 74.69% and 96.18%, respectively [97]. Wu et al. prepared polycaprolactone/ethylcellulose (PCL/EC) nanofiber membranes loaded with natamycin and trans-cinnamic acid using microfluidic blown film technology. The films demonstrated significant inhibitory activity against *Escherichia coli*, *Staphylococcus aureus*, and *Botrytis cinerea*, while exhibiting reduced fiber diameters, enhanced water vapor permeability, and improved antioxidant properties without compromising strawberry quality during storage [159].

Currently, the equipment used for microfluidic blow spinning is typically desktop machines, but factors such as their large size, high operating costs, and complex maintenance limit their widespread use [160]. The handheld spinning machine has advantages such as its light weight, compact size, and low cost, making it more convenient and flexible for practical applications. It is suitable not only for stable laboratory environments but also for outdoor or frequently shifting work scenarios, thereby broadening the application range of microfluidic blow molding spinning technology [161]. In addition, the hand-held spinning machine is equipped with an easy-to-use interface, operates with low power consumption, and does not generate pulse currents, even for people with no prior knowledge [162]. In recent years, some scholars have prepared nanofiber films using handheld spinning machines. For example, Guo et al. employed a handheld blown spinning device to in situ fabricate polycaprolactone/ethyl cellulose (PCL/EC) nanofiber membranes loaded with natamycin and trans-cinnamic acid on mango surfaces. After 9 days of storage, PCL/EC/Nt-p nanofiber membranes delayed the decline of antioxidant enzyme activities in treated mangoes, demonstrating an enhanced antioxidant capacity and suppressed metabolic processes [100]. It is worth noting that microfluidic technology is typically applied in the biomedical field, while post-harvest preservation technology for fruits is still in its infancy. Microfluidic chips designed for other applications may not be fully compatible with the preparation of fruit coatings.

In conclusion, electrospinning technology, blow spinning technology, and microfluidic spinning technology have immense potential in the field of fruit preservation coatings. However, they are still at the laboratory research stage, with a substantial amount of research focused on ectopic coating preparation, and only a limited number of studies have been conducted on in situ preparation directly on the fruit surface. These methods can be further optimized and improved for direct application on various fruit surfaces.

## 4. Conclusions and Future Trends

Overall, coating application technology has great potential in the post-harvest preservation of fruits. Currently, research is mainly focused on the preparation and characterization of coatings, as well as the testing of their preservation performance. However, there are several shortcomings: there is a lack of verification of coating stability under dynamic transportation conditions, a lack of assessment of suitability for secondary fruits, and a lack of research on consumer acceptance of coating appearance. Most of the research focuses on the preservation effect of the fruit in a static laboratory environment. However, one of the main reasons affecting the quality of the fruit after harvest and before sales is the mechanical damage during transportation. Research on major fruits such as apples, mangoes, and strawberries has been conducted in detail from various aspects such as coating composition and preparation technology, while research on secondary fruits remains limited [163]. The preservation effects of the same methods on major and secondary fruits differ, indicating a gap in the development of coatings for secondary fruits. Future researchers have ample opportunities to explore this area. In addition, some coatings produced in studies are white, which may be perceived as much less safe than transparent ones in the eyes of consumers.

As an efficient and environmentally friendly post-harvest preservation method, fruit coating technology is rapidly transitioning from traditional processes to multifunctional, intelligent, and lightweight solutions. Significant progress has been made in improving coating performance through material blending, structural reinforcement, and the addition of active molecules. Traditional coating technologies are susceptible to operational parameters and fruit surface characteristics, leading to poor coating uniformity and insufficient adhesion. Future research should focus on process improvements. Fiber coating formation technology is constrained by the low yield and high cost of nanofibers, hindering its commercialization. Further optimizing the process to achieve large-scale production is an urgent issue that needs to be addressed. Additionally, microfluidic spinning technology is still in its infancy in fruit preservation applications. Therefore, it is necessary to determine appropriate technical parameters, optimize the process, and combine it with active molecules to expand its application scope and versatility.

## Figures and Tables

**Table 1:** Advances in the application of traditional coating application techniques.

Object	Coating Method	Formulation	Coating Effects	References
Watermelon	Spraying	Sodium-alginate, pectin and calcium lactate	The shelf life of watermelon has been extended from 7 days to 12–15 days.	[42]
Orange	Spraying	Pea starch and guar gum	It is better than commercial wax in terms of extending shelf life (4 weeks refrigerated and 1 week on the shelf), maintaining organoleptic quality and inhibiting decay.	[43]
Banana	Spraying	Sucrose esters and rice starch	Effectively delayed ethylene biosynthesis and reduced respiration rate, extending the shelf life by 12 days compared to untreated controls.	[44]
Mango	Dipping	Bleached shellac, tannic acid, glycerol	TA-shellac extends shelf life by about 10 days compared to the control group, with significant improvement in browning inhibition, weight loss and flavor retention	[45]
Blueberry	Dipping	Gum Arabic, roselle flower extract, calcium chloride and glycerin	Gum Arabic coatings with roselle extract are more effective than plain ones in inhibiting microorganisms, reducing enzyme activity and anthocyanin degradation, increasing total phenolic content, and lowering the decay rate.	[46]
Tomato	Dipping	Mango kernel starch, Glycerin, sorbitol	The mango kernel starch coating delayed the ripening process of tomatoes up to 20 days during storage at 20 °C without negatively affecting post-harvest quality.	[47]
Longan	Dipping	0.5%, 1.0%, and 2.0% chitosan	Chitosan coating treatment reduced the respiration rate and oxidase activity, and the increased chitosan concentration effectively prolonged the storage time and quality of longan.	[48]
Papaya	Dipping	15%, 25%, and 50% aloe vera gel	Aloe vera coating effectively delays papaya ripening and extends shelf life, and can still be sold after 15 days of storage, and with better results than higher concentrations of aloe vera.	[49]
Lime	Dipping	Pectin, sorbitol, beeswax and monoglycerides	Compared with the control sample, the respiration rate of coated limes was inhibited, and fruit weight loss and firmness were reduced to a lower level.	[50]
Pear	Dipping	Chitosan, guar gum and lemon peel essential oil (1, 1.5, 2, 2.5, and 3.0%)	The guar gum and chitosan coating with lemon peel essential oil significantly reduces weight loss and improves firmness of pears when stored at 4 ± 2 °C for up to 45 days. In addition, the coating with 3% lemon peel essential oil had a higher overall acceptability.	[51]
Strawberry	Dipping	Lactobacillus lactis, Bacillus cinerea, chia seed mucus and gelatin	*Lactobacillus lactis* and kiwifruit mucilage improved the quality of strawberries after harvest, and the addition of 2–4% lactobacilli effectively improved the storage quality of strawberries.	[52]
Guava	Dipping	0.5%, 1.0%, and 2.0% chitosan	The chitosan coating helped to retard the ripening process of guava fruits during cold storage with better quality retention at a 2% concentration compared to 0.5% and 1%.	[53]
Zucchini	Brushing	Whey protein concentrate, Arabic gum, guar gum, glycerol, thyme essential oil	Coatings containing guar and gum Arabic (S) are rheologically superior to Tween 20 (T) coatings; T coatings are superior in reducing weight loss, retaining hardness, and maintaining sensory characteristics, and are more effective in extending shelf life.	[54]

**Table 2:** Comparative analysis of traditional fruit coating application methods.

Coating Method	Advantages	Disadvantages
Dipping	1. Can process large quantities of small fruits (such as blueberries and cherries) at a time, with high batch efficiency. 2. Can penetrate into the recesses of fruit stems, providing good coverage. 3. Only requires an open immersion tank, with simple operation and low cost.	1. Gravity causes more coating liquid to accumulate at the bottom of the fruit, resulting in uneven coating thickness. 2. Open slot dip coating easily leads to the accumulation of impurities, requiring frequent replacement of the coating. 3. Not suitable for large fruits such as watermelons and cantaloupes, which have long dripping times and low drying efficiency.
Vacuum dipping	1. In a vacuum environment, the coating can penetrate into micropores (such as the gaps between strawberry seeds), increasing the penetration area and enhancing the fresh-keeping effect. 2. Vacuum adsorption reduces dripping loss and minimizes coating waste.	1. Vacuum systems are expensive. 2. Vacuum environments can easily cause soft fruit cells to rupture, posing a risk of damage. 3. Precision control of parameters such as vacuum level and pressure is required, making operation difficult.
Brushing	1. No complicated equipment is required, and the cost is extremely low. 2. Local repairs (such as apple stem marks) can be made.	1. Prone to brush marks, bubbles, uneven coating, and poor decorative properties. 2. Repeated use of brush bristles may cause cross-contamination.
Spraying	1. The atomized spray provides comprehensive coverage and forms an even coating, suitable for smooth fruit surfaces. 2. Automated assembly line operation, fast speed, and adjustable spray heads for different fruits. 3. Good adaptability to curved and irregular surfaces.	1. High atomization loss and high paint loss. 2. Requires equipment such as spray guns and air compressors, resulting in high equipment costs. 3. Not suitable for porous fruits such as strawberries and bayberries, as excessive coating thickness can cause anaerobic respiration and produce an ethanol odor.

**Table 3:** Advances in the application of fiber deposition technology.

Object	Coating Method	Formulation	Coating Effects	References
Peach	Electrospinning	Zein, ethanol, and polyethylene oxide	The shelf life of peaches was extended by 4 days, and the fiber prepared from glutaraldehyde, corn protein, and PEO in a 1:5:5 ratio had a better preservation effect.	[93]
Apple	Electrospinning	Zein, ethanol, and curcumin	At 23 °C and 75% humidity, after 6 days, the diameter of the green mold lesions on the coated apples was reduced by nearly 50% compared to the uncoated apples.	[94]
Grapes and tomatoes	Electrospinning	Cinnamon bark oil, clove bud oil, cellulose acetate, dimethyl formaldehyde, and acetone	Using cellulose acetate nanofiber membranes loaded with 50% cinnamon bark oil and clove bud oil, the shelf life of fresh grapes and tomatoes was extended to 30 days at 4 °C, with minimal deterioration in physical and chemical properties.	[95]
Strawberry	microfluidic blow spinning	Polyvinyl alcohol, aqueous extract of acai pulp, cocoa shell, jabuticaba peel, and carrot pomace	Compared with the control fruit, jabuticaba peel and PVA as strawberry coatings resulted in less color change, reduced degradation of antioxidant activity and TPCs, and a 50% reduction in the incidence of rotten fruit during storage.	[96]
Apple	microfluidic blow spinning	Konjac glucomannan polyvinylpyrrolidone, ethanol, and Elderberry anthocyanin	KEA/PVP membranes exhibit excellent antioxidant properties, with DPPH and ABTS radical scavenging rates of 74.69% and 96.18%, respectively. Compared to the control group, fresh-cut apples showed the best preservation effect.	[97]
Mango	handheld microfluidic-blow-spinning	Polycaprolactone, ethyl cellulose, 2,2,2-trifluoroethanol, natamycin, and trans-cinnamic acid	PCL/EC/Nt-p nanofiber membrane treatment resulted in the smallest diameter of mango lesions and a 20% lower decay index compared to the untreated group. After 9 days of storage, the decline in antioxidant enzyme activity was delayed.	[98]
Cherry tomatoes	solution blow spinning	2,2,2-Trifluoroethanol, polycaprolactone, carboxymethyl chitosan, curcumin, thymol, Nisin, and natamycin	The film forms a barrier on the surface of cherry tomatoes, limiting water penetration, reducing fruit respiration, thereby reducing weight and hardness, and delaying the ripening of cherry tomatoes after harvest.	[92]

## Data Availability

No data was used for the research described in the article.

## Abbreviations

The following abbreviations are used in this manuscript:

WHO	World Health Organization
FAO	Food and Agriculture Organization of the United Nations
CuNPs	Copper nanoparticles
TEO	Thyme essential oil
ATP	Adenosine triphosphat
AcXETs	Annona cherimola Xyloglucan Endotransglycosylases
AcEXPs	Annona cherimola Expansins
AcPE	Annona cherimola Pectinesterase
DNA	DeoxyriboNucleic Acid
CS	Chitosan
TA	Tannic acid
DPPH	2,2-Diphenyl-1-picrylhydrazyl
TPC	Total phenolic compound
PS	Polystyrene
PMMA	Polymethyl methacrylate
PC	Polycarbonate
CA	Cinnamaldehyde
GA	Glutaraldehyde
PCL	polycaprolactone
PVA	Polyvinyl alcohol
PEGDA	Poly (ethylene glycol) diacrylate
PVB	Polyvinyl butyral
PSF	Polysulfone
PES	Polyethersulfone
PAAS	Sodium Polyacrylate
PVP	Polyvinylpyrrolidone
KGM	Konjac glucomannan
EA	Elderberry anthocyanin
KEA	Konjac glucomannan and elderberry anthocyanin mixing
EC	Ethylcellulose
LAG	Low-acyl gellan gum
PLA	Polylactic acid
GCMC	Gelatin carboxymethyl cellulose membranes
AM	Aegle marmelos
CS-TA/MXene	Tannic acid/MXene assembly with chitosan
PEO	Corn soluble protein containing hexanal–poly(ethylene oxide)
